# Supplementary material for: Development of a computer-aided design software for the quantitative evaluation of aesthetic damage
Source: PLoS One. 2019 Dec 18;14(12):e0226322. doi: 10.1371/journal.pone.0226322 (PMC6919621; doi:10.1371/journal.pone.0226322)
Supplement: S1 Code site — 1. (ZIP) [file pone.0226322.s001.zip › [EESC_jr][Projeto]Relatorio_07_21/reports/templates_zip/gentelella-master/vendors/Flot/examples/stacking/index.html]

Flot Examples: Stacking


## Stacking

With the stack plugin, you can have Flot stack the series. This is useful if you wish to display both a total and the constituents it is made of. The only requirement is that you provide the input sorted on x.

With stacking
Without stacking

Bars
Lines
Lines with steps

Copyright © 2007 - 2014 IOLA and Ole Laursen
